# Pietro Daniel Omodeo 2019: *Political Epistemology. The Problem of Ideology in Science Studies* und Monika Wulz, Max Stadler, Nils Güttler und Fabian Grütter (Hg.) 2021: *Deregulation und Restauration. Eine politische Wissensgeschichte*

**DOI:** 10.1007/s00048-022-00329-8

**Published:** 2022-03-21

**Authors:** Fabian Link

**Affiliations:** Bergische Universität Wuppertal, Wuppertal, Deutschland

**Pietro Daniel Omodeo 2019: *Political Epistemology. The Problem of Ideology in Science Studies*. **Cham: Springer, brosch., 155 S., 59,27 €, ISBN: 978-3-030-23122‑4.

**Monika Wulz, Max Stadler, Nils Güttler und Fabian Grütter (Hg.) 2021: *Deregulation und Restauration. Eine politische Wissensgeschichte*. **Berlin: Matthes & Seitz, brosch., 270 S., 22,00 €, ISBN: 978-3-7518-0322‑9.

2010 forderte Volker Roelcke eine stärkere Einbeziehung des Politischen in die historische Wissenschaftsreflexion (*Berichte zur Wissenschaftsgeschichte*, Bd. 33, S. 176–192). Für ein angemessenes Verständnis historischer Phänomene sei „die systematisierte Einbeziehung und Reflexion der politischen Dimension bei der Produktion von neuem Wissen in den Wissenschaften“ unverzichtbar (176). Die beiden hier zu besprechenden Publikationen – eine Monografie von Pietro Daniel Omodeo und ein Sammelband, herausgegeben von Monika Wulz, Max Stadler, Nils Güttler und Fabian Grütter – lösen diese Forderung programmatisch ein. Die Autor*innen gehen ganz unterschiedlich an das Projekt einer politisch-historischen Epistemologie beziehungsweise politischen Wissensgeschichte heran. Während Omodeo die historische Epistemologie nach Hans-Jörg Rheinberger mit einer habermasianischen Ideologiekritik und der Herstellung von Diskurshegemonien nach Antonio Gramsci sowie mit Marx’ Kritik der politischen Ökonomie kombiniert, versammelt der Band von Wulz et al. Texte über Bücher und Aufsätze, die das neoliberale Bewusstsein begründeten, wie es sich in Westeuropa und Nordamerika, aber auch im Globalen Süden und in Asien seit Mitte der 1970er Jahren entwickelte und etablierte. Beiden Büchern ist die Auffassung gemein, dass es eine nichtpolitische und nichtideologische Wissenschaft und Wissensproduktion nicht gibt und niemals gab. Dass nicht wenige Wissenschaftler*innen – etwa während des Kalten Krieges in den westlichen Ländern – gerade dies behaupteten, ist insbesondere für Omodeo ein Aufhänger seiner politisch-epistemologischen Kritik. Auf das Ende des Kalten Krieges folgten Triumphgeschichten, die vom Ende der Ideologien (und der Geschichte) schwadronierten, was mit der seit den frühen 1980er Jahren in den USA und Großbritannien, etwas später und weniger radikal auch in der Bundesrepublik vollzogenen „konservativen Tendenzwende“ korrespondierte. Doch die 1980er und 1990er Jahre waren alles andere als unpolitisch oder entideologisiert – im Gegenteil bereiteten sie den Boden für die heutige *post-truth*- und *alternative-facts*-Ära.

Das Politische begreift Omodeo als Ideologie, der er zwei reflexive Funktionen zuschreibt: einerseits eine selbstreflexive im Sinne einer adäquateren Vergegenwärtigung unserer eigenen Standpunkte, denn Erkenntnistheorie ist für ihn nicht bloß analytische Wissenschaftsphilosophie, sondern muss eine selbstkritische Reflexion von Ideologie beinhalten, darf weder Positivismus noch postmoderner Wissenschaftszynismus sein (22, 149). Andererseits möchte er mit Gramscis Hegemonieansatz Ideologie als wissenschaftliche Haltung positiv aufladen, indem er diese als auf die Verpflichtung für die Würde des Menschen, Emanzipation und Situierung der eigenen Position, höhere Gewichtung der Perspektive und Position der Subalternen sowie eine kapitalismuskritische Einstellung ausgerichtet konzeptualisiert (151–154). Omodeo folgt dabei Gramscis Vorstellung von Objektivität, die jener als eine durch die Subalternen erkämpfte, auf die wirklich existierenden Verhältnisse reflektierende Position begreift (137). Es geht ihm um die Konzeption einer radikaldemokratischen und emanzipierten Wissenschaft, in der den Wissenschaftshistoriker*innen die Rolle zivilgesellschaftlich engagierter und politisch intervenierender Intellektueller zukommt (141). Basis einer so gearteten Wissenschaftsforschung ist die integrierte Behandlung von Ökonomie und Politik bei der Analyse der Wissenschaften (117). Dabei gewichtet Omodeo die Rolle der Wissenschaften als die Gesellschaft steuernder Bereich ausgesprochen hoch: „Science ist the most powerful instrument of hegemony, control, and guidance of modern societies.“ (153).

In den fünf Kapiteln des Hauptteils verfolgt Omodeo konsequent diese doppelte Perspektive: einerseits eine politische Reflexion der Rolle von Ideologie in der Geschichte der Wissenschaften im 20. Jahrhundert, andererseits die kritische Reflexion darüber, wie Ideologiekritik geleistet und Ideologie-Hegemonie nach Gramsci hergestellt werden kann. In Kapitel 2 richtet Omodeo die *Science Studies* als politische Epistemologie neu aus, Kapitel 3 verklammert Modernität und die Logik von Wissenschafts- und Technologieentwicklung. Kapitel 4 – der für Wissenschaftshistoriker*innen wohl spannendste Teil – stellt die Verwerfungen und Verflechtungen sozialistisch-kommunistischer und kapitalistisch-liberaler Wissenschaftsreflexionen während des Kalten Krieges dar. Mit Rekurs auf Marx versucht Omodeo in Kapitel 5 dann die Grundlage einer soziopolitischen Wissenschaftsgeschichte zu skizzieren, auf der die in Kapitel 6 präsentierte Hegemonieherstellung einer emanzipierten Wissenschaftsideologie nach Gramsci aufbauen soll. Kritisch anzumerken ist, dass Omodeos Konturierung von linken, rechten sowie liberalen Positionen an manchen Stellen schematisch erscheint. So folgt er etwa der Argumentationslinie, internalistisch-idealistische Wissenschaftsforschung sei affirmativ, wogegen externalistisch-historisch-materialistische Wissenschaftsforschung kritisch sei (81–82).

Die Autor*innen des Bands von Wulz et al. begründen dagegen ihren kritisch-reflexiven Standpunkt aus dem historischen Material selbst heraus, indem sie unter den Begriffen Deregulation und Restauration Gründungstexte des Neoliberalismus und teilweise des Postmodernismus durchleuchten. Jakob Tanner analysiert unter der Kapitelüberschrift „Ordnung“ Wilhelm Röpkes *Internationale Ordnung* von 1945, Monika Dommann erforscht unter dem Schlagwort „Informelle Ökonomie“ Hernando de Sotos *El otro sendero* aus dem Jahr 1986 und Laura Rischbieter untersucht unter dem Titel „Märkte“ Alan Greenspans *The Age of Turbulence* von 2007, um nur drei der insgesamt 15 ausgesprochen lesenswerten Beiträge zu nennen. Die Auswahl der verschiedenen Autor*innen und Texte erscheint zwar nicht willkürlich, aber doch etwas impressionistisch, zumal methodisch nicht begründet wird, weshalb Röpke und nicht Friedrich A. von Hayek, Niklas Luhmann und nicht Renate Mayntz oder Elisabeth Noelle-Neumann und nicht Erwin K. Scheuch als Referenzautor*innen ausgewählt wurden. Angesichts dessen, dass hier eine Zeit untersucht wird, die von der „neuen Unübersichtlichkeit“ (Jürgen Habermas) geprägt war, stellt der Band aber auch keinen Anspruch auf Vollständigkeit.

Ausgesprochen beeindruckend ist das hohe Reflexionsniveau der Beiträge, weil die Autor*innen den ambivalenten Charakter neoliberal-postmoderner Denkhaltungen herausarbeiten. Dies soll abschließend anhand eines der Beiträge gezeigt werden – der unter dem Stichwort „Lokales Wissen“ von Fabian Grütter, Nils Güttler, Max Stadler und Monika Wulz angestellten Untersuchung des Buchs *Local Knowledge* von Clifford Geertz von 1983, das „eine Pluralisierung und Dezentrierung ‚westlicher‘ Maßstäbe“ leistete und damit „eine groß angelegte Deregulation der herkömmlich-westlichen Wissensordnung“ vollzog (164). In etwa derselben Zeit kamen auch Neurechte wie der „Nationalrevolutionär“ Henning Eichberg auf die Idee, auf lokales Wissen zu rekurrieren mit dem Ziel, die eigene nationale Kultur zu restaurieren und gegen die „Amerikanisierung“ in Stellung zu bringen. Aber auch Geertz’ Buch stellte eher eine Umcodierung eines antikommunistischen und eurozentrischen Modernisierungsdiskurses dar und unterlag damit einer gewissen Ambivalenz. In den 1950er und frühen 1960er Jahren hatte er schließlich noch die Ansicht vertreten, Armut sei kein Resultat kolonialer Machtverhältnisse, sondern Effekt kultureller Rückständigkeit (167). Die Autor*innen machen deutlich, dass seine Vorstellung über lokales Wissen selbst historisch-genealogisch lokalisiert werden muss, denn Geertz schrieb in Indonesien, wo er sich in den 1950er Jahren aufhielt, anders als in Marokko, wo er in den 1960er Jahren einen „Pilgerort derjenigen Amerikaner*innen, die die repressiven Entwicklungen der McCarthy-Ära mit Sorge zur Kenntnis nahmen“, vorfand (168).

Beide Werke sind uneingeschränkt zur Lektüre zu empfehlen und es ist zu hoffen, dass die von den Autor*innen vorgestellten Perspektiven in zukünftigen Projekten weiterentwickelt werden. Eine noch detaillierter zu beantwortende Frage wäre, auf welche Weise Neoliberalismus und Postmodernismus genau zusammenhängen und welche Bedeutung dies für die historische, philosophische und soziologische Wissenschaftsforschung bis heute hat.

